# Is liquid biopsy mature enough for the diagnosis of Alzheimer’s disease?

**DOI:** 10.3389/fnagi.2022.977999

**Published:** 2022-08-05

**Authors:** Xun Gong, Hantao Zhang, Xiaoyan Liu, Yi Liu, Junlin Liu, Funmilayo O. Fapohunda, Peng Lü, Kun Wang, Min Tang

**Affiliations:** ^1^Department of Rheumatology and Immunology, Affiliated Hospital of Jiangsu University, Zhenjiang, China; ^2^School of Life Sciences, Jiangsu University, Zhenjiang, China; ^3^Institute of Animal Husbandry, Jiangsu Academy of Agricultural Sciences, Nanjing, China; ^4^Children’s Center, The Affiliated Taian City Central Hospital of Qingdao University, Taian, China

**Keywords:** Alzheimer’s disease, liquid biopsy, blood, CSF, biomarkers

## Abstract

The preclinical diagnosis and clinical practice for Alzheimer’s disease (AD) based on liquid biopsy have made great progress in recent years. As liquid biopsy is a fast, low-cost, and easy way to get the phase of AD, continual efforts from intense multidisciplinary studies have been made to move the research tools to routine clinical diagnostics. On one hand, technological breakthroughs have brought new detection methods to the outputs of liquid biopsy to stratify AD cases, resulting in higher accuracy and efficiency of diagnosis. On the other hand, diversiform biofluid biomarkers derived from cerebrospinal fluid (CSF), blood, urine, Saliva, and exosome were screened out and biologically verified. As a result, more detailed knowledge about the molecular pathogenesis of AD was discovered and elucidated. However, to date, how to weigh the reports derived from liquid biopsy for preclinical AD diagnosis is an ongoing question. In this review, we briefly introduce liquid biopsy and the role it plays in research and clinical practice. Then, we summarize the established fluid-based assays of the current state for AD diagnostic such as ELISA, single-molecule array (Simoa), Immunoprecipitation–Mass Spectrometry (IP–MS), liquid chromatography–MS, immunomagnetic reduction (IMR), multimer detection system (MDS). In addition, we give an updated list of fluid biomarkers in the AD research field. Lastly, the current outstanding challenges and the feasibility to use a stand-alone biomarker in the joint diagnostic strategy are discussed.

## Introduction

Alzheimer’s disease (AD) is a neurologic condition in which the brain shrinks and brain cells necrotize, which is characterized by a progressive loss of cognitive, behavioral, and social abilities (Soria Lopez et al., 2019). In the United States, AD is recognized as the most common cause of dementia, which affects about 5.1 million people aged 65 and up, impairing their ability to work and live independently (Lane et al., 2018). Memory loss, intellectual disability, various cognitive abnormalities, and disorientation are among the symptoms of AD, and risk factors include age, sex, down syndrome, familial inheritance, and poor sleeping habits (Silva et al., 2019). Furthermore, the death rate from AD has been growing substantially, with a 71% increase from 2000 to 2013 (Cass, 2017; Tiwari et al., 2019). In the regions of the brain affected by this disease, the accumulation of intracellular neurofibrillary tangles (NFT) and extracellular amyloid plaques was found. These plaques are mainly comprised of neurotoxic amyloid-beta protein 40 (Aβ40) and amyloid-beta protein 42 (Aβ42), which would have a devastating effect on neuronal cells (Hodson, 2018; Breijyeh and Karaman, 2020). Though AD remains a major public health problem, there are only two classes of pharmacologic therapy available for treating patients with AD, which include inhibitors to cholinesterase enzyme and antagonists to *N*-methyl-D-aspartate (NMDA). It was proved that these two classes can only be used to treat the symptoms of AD but not to cure it (Chu, 2012; Birks and Harvey, 2018; Atri, 2019). Due to the difficulty of treating AD, special attention should be paid to developing methods for accurate and timely diagnosis.

Normally, the diagnosis of AD is based on clinical evaluation, with several tests required for a conclusive diagnosis, including neuropsychological examination, magnetic resonance imaging (MRI) or computed tomography (CT) for neurons, and serum vitamin B12 level test (Hane et al., 2017). However, early diagnosis of AD is also complicated since early symptoms are shared by several diseases with similar neuropathological features (Weller and Budson, 2018). The identification of measurable, non-invasive, and reliable biomarkers for AD early diagnosis must help address this problem. Biological markers, or biomarkers, are widely used in human pathology for both diagnosis and disease monitoring. They are parameters determined by biological methods and primarily used to determine whether a certain disease exists or not, as well as the likelihood of contracting it (Wu and Qu, 2015). Actually, in the fields of lung cancer, colorectal cancer, and pancreatic, research has already been conducted to discover liquid biopsy biomarkers for cancer prognosis (Vaidyanathan et al., 2018). Unlike tumor tissue biopsy, liquid biopsy is a non-invasive detection method that can enable researchers to get information from body fluid samples by sampling and analyzing these samples and monitoring the patient condition in real-time (Poulet et al., 2019). In addition, tumor tissue biopsy is rather difficult to conduct in several cases. Because liquid biopsy is easier, quicker, and less painful than conventional biopsy, it is a promising alternative to existing surgical biopsies (Mader and Pantel, 2017).

Given the breakthrough nature of liquid biopsy in Oncology, there is a promising rationale for trans-fertilization and validation of blood-based liquid biopsy in AD (Hampel et al., 2019). Since there are no specific signs or symptoms associated with AD, the discovery of blood biomarkers for AD allows for a less invasive and more precise diagnosis, which may aid in identifying patients at risk before clinical symptoms and complications occur and defining disease stages (Lashley et al., 2018). The liquid biopsy would be especially useful in identifying people with preclinical AD, namely those with AD neuropathology but no clinical symptoms (Markesbery et al., 2006). In this review, we introduced liquid biopsy and the role it plays in disease diagnosis. We also reviewed diversified liquid biopsy tools which sensitively reflect a person’s health status and would be used for AD diagnosis. Finally, we discussed challenges and envisioned the future of liquid biopsy.

## What is liquid biopsy?

**FIGURE 1:**
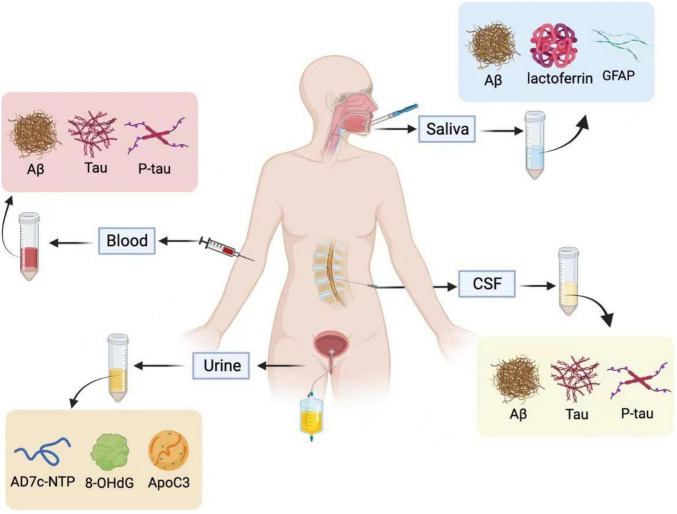
Saliva, blood, cerebrospinal fluid (CSF), and urine are used for Alzheimer’s fluid biopsy. In the CSF and blood, Aβ, T-tau, and P-tau are used as biochemical indicators for the early diagnosis of Alzheimer’s disease. The specific biomarkers in the urine are AD7c-NTP, 8-OHdG, and ApoC3, and salivary biomarkers are Aβ, Lactoferrin, and GFAP. Illustrations were generated using BioRender.

Biopsy is a set of processes that include removing cells or tissues from the primary or metastatic mass and analyzing these samples to find out the causes of patients (De Rubis et al., 2019). In a traditional biopsy, the acquisition of specimens requires a biopsy needle or surgical procedure (see Figure 1). Given their invasive nature, tissue biopsy presents several limitations, which include patient risk, procedural costs, and invasive testing (Chen et al., 2020). In this regard, liquid biopsy can overcome these disadvantages and provide patients with a minimally invasive approach capable of cancer diagnosis (Smania, 2020).

Liquid biopsy is defined by the National Cancer Institute (NCI) as “a test done on a sample of blood to look for cancer cells from a tumor that are circulating in the blood or for pieces of DNA from tumor cells that are in the blood” (Underwood et al., 2020). Though blood is most commonly used, the use of other kinds of body fluids such as CSF, urine, saliva, and stool are also involved in liquid biopsy (Blanco and Wolfgang, 2019). Compared with samples taken from the primary tumor, body fluid reflects a much broader range of tumor properties. The tumor circulome is defined as a portion of circulating components shed from tumor tissue that can be collected from body fluid (Mader and Pantel, 2017). Analysis of elements of tumor circulome including tumor circulome comprises of circulating tumor cells (CTCs), circulating tumor DNA (ctDNA), circulating tumor RNA (ctRNA), and extracellular vesicles (EVs) serves as a superb way for screening, diagnosis, and prognosis of many cancer types (Rohanizadegan and Kulkarni, 2018; Poulet et al., 2019). The number of free CTCs and EVs can both inform the presence of malignant disease and tumor burden (Kloten et al., 2019). Similarly, ctDNA can be used for the early detection of cancer. Besides, nearly all known classes of ctRNA have the potential to serve as biomarkers in cancer diagnosis (Pessoa et al., 2020).

The idea of liquid biopsy is not restricted to oncology but applies to AD diagnosis. Though a definitive method of AD diagnosis is brain tissue evaluation, it is impossible to take samples of living patients’ brain tissue (Weller and Budson, 2018). Biomarkers derived from CSF analysis have been used for *in vivo* AD detection (Hampel et al., 2021). Because it is generally believed that the onset of AD relates to the deposition of amyloid-β (Aβ) in extracellular plaques, aggregation of tau into neurofibrillary tangles in neurons, and abnormal phosphorylation of tau, the measurements of Aβ1–42, hyperphosphorylated tau, and total tau proteins are options for AD early diagnosis (Hersi et al., 2017; Park et al., 2020). However, this method is risky and inconvenient due to the invasive nature of its lumbar puncture procedure (Li et al., 2019). It often takes weeks to obtain final results due to the deficiency of laboratory facilities, which makes CSF analysis unsuitable for broad clinical implementation (Delgado-Peraza et al., 2021). Blood-based liquid biopsy is an ideal substitute for CSF-based liquid biopsy since it is non-invasive and cost-effective (Abdel-Haq, 2020).

Blood-based liquid biopsy for preclinical or clinical AD diagnosis is a multistep work-up (Baldacci et al., 2019). For example, EVs are regarded as attractive tools for non-invasive diagnosis since they are stable and contain diverse molecules from their parental cells. To use blood-derived EVs to determine if a person suffers from AD, blood samples should be obtained through venipuncture, and EVs should be isolated from blood (Mustapic et al., 2017). The major isolation strategies include ultracentrifugation, polymer precipitation, ultrafiltration, size-exclusion chromatography, affinity isolation, and microfluidics-based techniques. Finally, clinicians can gain a view of an individual’s health status by detecting the neurogranin level in blood-derived EVs, since it is downregulated in AD patients (Liu J. et al., 2021).

While liquid biopsy is a minimally invasive and cost-effective method that could be used to detect disease before patients have symptoms, many problems still need to be solved before liquid biopsy become a great alternative to tissue biopsy (De Rubis et al., 2019; Smania, 2020). First and foremost, the sensitivity and specificity of liquid biopsy assays need to be refined (Underwood et al., 2020). Meanwhile, protocols, pre-analytical, and analytical procedures need to be standardized (Blanco and Wolfgang, 2019). These procedures may include sample collection, liquid biopsy components isolation, analysis, data organization, and sharing (Poulet et al., 2019).

## The cutting-edge liquid biopsy technologies for Alzheimer’s disease

Blood sampling may be preferable to CSF sampling due to its convenient, inexpensive, safe, and minimally invasive nature, but measuring brain disease biomarkers in the blood presents a variety of challenges. Since the blood-brain barrier prevents molecules from freely passing between the central nervous system (CNS) and blood, brain-derived biomarkers are usually found in low concentrations in blood (Lista et al., 2013). Also, biomarkers linked to AD pathology are rarely concentrated in non-cerebral tissues, making blood measurements challenging. Even though certain biomarkers linked to Alzheimer’s disease pathology are expressed in non-cerebral tissues, their measurement in the blood could be muddled (Blennow and Zetterberg, 2018a; Liu L. et al., 2021). To solve this problem, several ultra-high-sensitivity technologies capable of quantifying concentrations of biomarkers not only in CSF but also in blood have been developed. These innovative technologies can be applied to the early stage diagnosis of AD (Kulichikhin et al., 2021).

### Stable isotope labeling kinetics

The Stable Isotope Labeling Kinetics (SILK) technique has been widely used to measure the protein turnover in human plasma and CSF such as three Aβ peptides (Aβ38, Aβ40, and Aβ42), tau and/or p-tau, superoxide dismutase 1 (SOD1) and three ApoE isoforms (ApeE2, ApoE3, ApoE4) (Crisp et al., 2015; Paterson et al., 2019; Dincer et al., 2020; Li et al., 2021; Elbert et al., 2022). In the process of using this technique, the different Aβ were immunoprecipitated simultaneously via monoclonal anti-Aβ mid-domain antibodies and later quantified by the liquid chromatography mass spectrometry (LC-MS/MS). In amyloid positive individuals, the Aβ42 concentrations and Aβ42/Aβ40 concentration ratios are significantly lower than those in amyloid negative individuals, which is consistent with that in CSF. Though the differences in plasma Aβ42/Aβ40 ratios between amyloid positive and negative individuals are of lesser magnitude compared to CSF, the LC-MS/MS ensures specificity and precision of results, which makes SILK a reliable tool for AD diagnosis (Potter et al., 2013; Baker-Nigh et al., 2016; Ovod et al., 2017). In the early stages, Basak et al. (2012) successfully measured the clearance rates of ApoE and Aβ in the mouse brain pulse-labeled by ^13^C_6_-leucine. Combining with the nanoscale secondary ion mass spectrometry (NanoSIMS) imaging technique, Wildburger et al. (2018) proposed a strategy termed SILK-SIMS to detect plaque dynamics in human AD brains. On the cellular level, the SILK technique can also be employed to measure tau kinetics with multiple isoforms and fragments in the induced pluripotent stem cell (iPSC)-derived neurons (Sato et al., 2018). The big limitation of this technique is the high labeling costs.

### Fully automated assays

With the rapid development of technology, fully automated immunoassays such as Elecsys immunoassays (Roche Diagnostics) are now available to measure the CSF or plasma biomarkers with high precision and stability (Asberg et al., 2019; Blennow and Zetterberg, 2019). Using this newly developed Elecsys immunoassay (Palmqvist et al., 2019) examined the Aβ42, Aβ40, total levels of tau protein (T-tau), and phosphorylated tau (P-tau) in the CSF and plasma in two cohorts. It was found that plasma Aβ42 and Aβ40 detected by the fully automated Elecsys platform could be used to predict brain Aβ positivity. Furthermore, additional analysis of other blood biomarkers such as ApoE genotype, T-tau, and neurofilament light chain (NFL) can increase the accuracy of Aβ status prediction (Palmqvist et al., 2019). Meanwhile, the fully automated immunoassay system termed High Sensitivity Chemiluminescence Enzyme Immunoassay (HISCL™ series) based on the plasma biomarkers has been emerging in the diagnosis market (Goto et al., 2019; Miura et al., 2020; Watanabe et al.; Noda et al., 2021).^1^ Yamashita et al. (2021) declared that they had developed Aβ40 and Aβ42 immunoassays using the HISCL™ series, which has higher reproducibility, wider dynamic range, and less reaction time compared with the conventional enzyme-linked immunosorbent assays (ELISA). Hence, the fully automated plasma assays as a high-throughput and highly reliable screening tool might be used for clinical AD trials, which would help the patients get rid of unnecessary lumber punctures and save the expanse of examination.

### Superconducting quantum interference device-based immunomagnetic reduction (SQUID IMR)

The immunomagnetic reduction (IMR) biomarker detection system was established in the early 2000s (Yang et al., 2017). Magnetic nanoparticles used in IMR are particles whose core comprises Fe3O4 and surface consists of bio-probes and hydrophilic surfactants. The phosphate-buffered saline (PBS) solution where these particles are suspended presents a magnetic property under external alternative-current (ac) magnetic fields, which is termed as *x*ac. When magnetic nanoparticles and target molecules bond together, these particles will become larger and result in a reduction in *x*ac. IMR (%) is the percentage of the reduction in magnetic signals during immune reaction divided by magnetic signals before immune reaction. The reduction in *x*ac could be quite low, which requires magnetic sensors with high sensitivity (Lue et al., 2019). The superconducting-quantum interference-device (SQUID) magnetometer is capable of measuring IMR. The biomarker concentrations can be calculated from IMR via standard curves. Because SQUID IMR can be applied to quantify molecules of any size in human plasma, researchers have utilized this technology to predict the progression of dementia in high-risk groups. They found that the plasma Aβ42 level is an intriguing index for early detection of cognitive decline in patients with Down syndrome, stroke, or amnestic mild cognitive impairment (aMCI). In addition, the SQUID IMR technology is suitable for predicting Aβ status and brain atrophy (Yang et al., 2020).

### Multimer detection system—Oligomerized amyloid beta (MDS-OAβ)

Meng et al. (2019) and Youn et al. (2019) presented the association between Aβ oligomerization in blood and AD neurodegeneration. An et al. (2017) found that Aβ oligomerization was significantly higher in AD patients than in normal people. Plus, the high correlations between Aβ oligomerization levels and other AD biomarkers, such as phosphorylated tau protein (pTau), amyloid positron emission tomography (PET) imaging, CSF biomarkers for Aβ42, total tau, and brain magnetic resonance imaging (MRI) were confirmed in many studies (Choi et al., 2021; Cheng et al., 2022).

**FIGURE 2:**
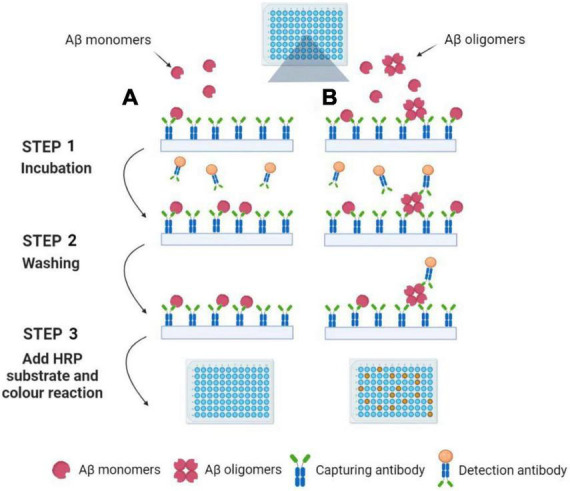
Detection of Aβ oligomers using the Multimer Detection System. **(A)** Aβ oligomers do not exist in blood plasma samples. Aβ monomers can be captured by an antibody attached to the ELISA plate’s surface. After incubation and washing, the detection antibodies are not present in the well because the capture antibodies have already occupied the single epitope. Finally, the color reaction can be not observed with the addition of HRP substrate. **(B)** Aβ oligomers exist in blood plasma samples. Aβ monomers and oligomers can be captured by an antibody attached to the ELISA plate’s surface. After incubation and washing, the detection antibodies are still present in the well because the Aβ oligomers have a lot of epitopes. Finally, the color reaction can be observed with the addition of HRP substrate. Illustrations were generated using BioRender.

Multimer detection system (MDS) is a traditional analytical platform based on modified ELISA to measure Aβ oligomerization tendency (An et al., 2010; Lim et al., 2015; Pyun et al., 2021). The epitope-overlapping detection antibodies applied in MDS are specifically designed for the N-terminus of Aβ for the selection of oligomerized formation over monomeric formation (Wang et al., 2017). Since the Aβ monomers have only one epitope available, once it has associated with a capturing antibody, it has no exposed epitopes to react with the antibodies (see Figure 2). Rather, the Aβ multimers with overlapping epitopes which bind the antibodies can be easily detected (An et al., 2010). Because of the ability to screen multimers, MDS has been utilized to differentiate the oligomeric Aβs (OAβ) from the monomeric Aβ in blood. Specific antibodies are used in MDS, and the level of OAβ can be obtained by using a spectrophotometer. As the OAβ test has the potential for distinguishing between AD patients and healthy controls, MDS would be a promising method for AD diagnosis, for example, AlphalLISA assay and the Blood™ OAβ test (PeopleBio Inc., South Korea) (Lee et al., 2020; Babapour Mofrad et al., 2021; Dominguez et al., 2022).

### Single molecule array

**FIGURE 3:**
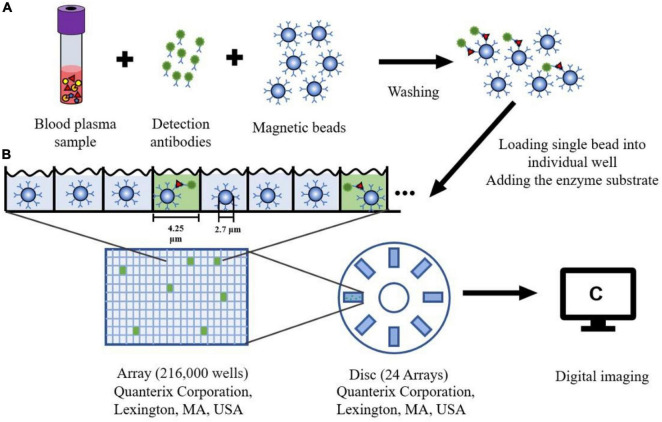
The schematic diagram of Single Molecule Array. **(A)** Blood plasma sample is mixed with the magnetic microbeads (2.7 μm) with capture antibodies and detection antibodies. After washing the beads three times, the protein can be captured specifically bound by the antibody. **(B)** Each Single magnetic bead is loaded into one well (4.25 μm diameter, 3.25 μm depth) of the array (216,000 wells). The substrate for the enzyme is added to the well sealing with oil. **(C)** After fluorescence detection of each well, a single molecule signal image can be obtained on this array.

The single molecule array (Simoa), also known as the single molecule enzyme-linked immunosorbent assay, is a bead-based immunoassay with the ability to detect protein biomarkers in blood at subfemtomolar concentrations (Cohen and Walt, 2017). The specific steps of this approach are as follows: Firstly, paramagnetic beads coated with capture antibodies are added to a sample. The amount of these capture beads is far beyond the number of target analytes contained in a sample so that each capture bead binds to zero or one target analyte. Next, each capture bead is incubated with a biotinylated detection antibody and an enzyme, streptavidin-β-galactosidase (SβG), to form an enzyme-labeled immunocomplex. Thirdly, the beads are suspended in a fluorogenic substrate solution and then loaded into an array (see Figure 3). Finally, fluorescence images of the assay are utilized to determine the concentration of a protein biomarker (Cohen et al., 2018, 2020).

Many studies verify that the Simoa assays examining different P-tau isoforms (P-tau181, P-tau217, P-tau231) have high accuracy and specificity to detect early Alzheimer’s pathology (Barthelemy et al., 2020b; Karikari et al., 2020, 2021a, 2021b; Palmqvist et al., 2020; Suarez-Calvet et al., 2020; Thijssen et al., 2020; Janelidze et al., 2021; Keshavan et al., 2021; Rauchmann et al., 2021; Ashton et al., 2022). Barthelemy et al. (2020a) and Janelidze et al. (2020b) declared that P-tau217 has higher accuracy than P-tau181 in CSF. Also, Palmqvist et al. (2020) believed that P-tau217 was a better biomarker than P-tau181 in a comparative plasma study, while it was not reproductive with an updated assay (Brickman et al., 2021). In an overall study, Bayoumy et al. (2021) constructed another comparative plasma study to test three P-tau181 assays (Eli Lilly, ADx, and Quanterix), a P-tau217 assay (Eli Lilly), and two P-tau231 assays (ADx, Gothenburg). All of the six Simoa assays showed robust clinical performance and high-sensitive. Notably, Lantero-Rodriguez et al. (2021) developed a new immunoassay targeting P-tau235 for staging preclinical AD. Currently, the Simoa NF-light Advantage Kit comes on the market, which quantifies the Neurofilament Light (Nf-L) in serum and plasma with high sensitivity (Hendricks et al., 2019; Ashton et al., 2020; Benussi et al., 2020; Manouchehrinia et al., 2020). The blood Nf-L level is becoming a supplemental biomarker of AD, there is little doubt that Simoa is a practical tool for AD diagnosis (Illan-Gala et al., 2021; Leuzy et al., 2021; Zetterberg and Blennow, 2021).

### Immunoprecipitation–mass spectrometry

**FIGURE 4:**
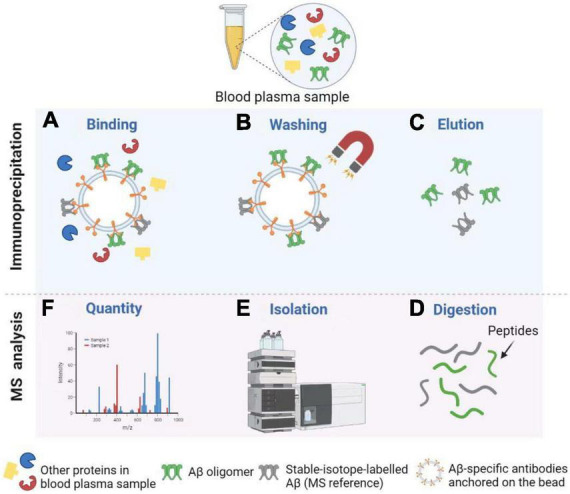
Detection of Aβ oligomers using Immunoprecipitation and Mass Spectrometry (IP-MS). **(A)** Aβ oligomers and stable-isotope-labeled Aβ (MS reference) can be captured by Aβ-specific antibodies anchored on the bead. **(B)** After washing, other proteins in the blood plasma sample are removed from the solution. **(C)** Next, Aβ oligomers and MS reference can be separated from the bead for further MS analysis. **(D,E)** After enzymatic digestion, peptides are isolated by High Performance Liquid Chromatography (HPLC). **(F)** Finally, MS profiles can be obtained to carry out the Aβ oligomer quantity. Illustrations were generated using BioRender.

Immunoprecipitation combined with matrix-assisted-laser desorption/ionization time of flight mass spectrometry, or called immunoprecipitation–mass spectrometry (IP–MS) for short, to examine the truncated Aβ peptides is an approach for characterizing protein and peptide mixtures in body fluid (Portelius et al., 2006; Guard et al., 2019). During the IP, target proteins or peptides at low concentrations are enriched using antibodies (see Figure 4). Then MS allows an accurate analysis of levels of these proteins or peptides (Uljon et al., 2000; Campbell et al., 2021). Richard et al. (2019) proposed a simplified ad detailed protocol for robust immunoprecipitation of Aβ before mass spectrometric detection. They clarified that the employment of murine monoclonal and rabbit polyclonal antibodies can yield reproducible and high-resolution pictures with low background noise (Richard et al., 2019). The IP–MS method is commonly used to analyze the Aβ38, Aβ40, and Aβ42 levels in plasma, and companies with SILK method in CSF (Ovod et al., 2017). To reduce the amount of lab workload and accelerate the SILK studies, Mawuenyega et al. (2013) developed an efficient and specific quantitative isoform characterization and kinetics method. Plenty of studies adopting the IP-MS method elucidated that plasma Aβ42 concentration and Aβ42/Aβ40 rate are promising biomarkers for predicting Aβ status in individuals (Portelius et al., 2006, 2015; Ovod et al., 2017; Cheng et al., 2022). Furthermore, immunoprecipitation together with SampleStream mass spectrometry (SampleStream-MS) was established. The streamlined workflow could be used to profile protein products of genes efficiently (Santos Seckler et al., 2021).

In sum, a huge development has taken place in the liquid biopsy platform in the past two decades. Researchers used to apply the traditional ELISA to detect and measure core CSF biomarkers, including Aβ42, T-tau, and P-tau (Yoo et al., 2018). While CSF possesses the advantage of being continuous with the brain extracellular fluid, there is little doubt that blood is a more ideal subject for liquid biopsy. However, studying biomarkers in the blood is more challenging than in CSF, for blood only contains a tiny amount of brain protein (Blennow and Zetterberg, 2018b). The advancements in the field of ultra-high-sensitivity technologies provided a solution to this problem. Although these screening tools can accurately quantify biomolecules at ultralow levels, some shortcomings limit their utility in AD biomarker studies (Hampel et al., 2021). For example, due to the spectral overlap of fluorescent dyes, they cannot be used for the simultaneous detection of multiple analytes. In addition, interactions between non-target molecules during the assay can generate erroneous signals, thereby having a negative effect on their sensitivity and specificity (Cohen and Walt, 2017).

## Histologic origin used in liquid biopsy and biological markers involved

A biological marker, or biomarker (Cedazo-Minguez and Winblad, 2010), is an indication of normal biological processes, pathogenic processes, or pharmacological responses to a therapeutic intervention that is objectively assessed and evaluated. The most important factors that determine a biomarker’s diagnostic utility are its sensitivity, specificity, and ease of use. Since the cognitive symptoms of AD are often diffuse and overlap with other disorders, and the clinical progression is intermittent and variable even among patients with the same disease, biomarkers for AD are extremely important. Biomarkers representing various forms of pathophysiology in the brain can be used for clinical diagnosis, particularly in the early stages of the disorder, to forecast progression, to observe the effects of novel drug candidates in clinical trials, and finally, to further our understanding of the disease’s pathogenesis in clinical science (Table 1, Blennow et al., 2010). Aiming to gain an insight into biomarkers for the early diagnosis of AD, molecules in CSF and blood have been intensively studied.

**TABLE 1:** Statistics of biomarkers in saliva, CSF, blood, Serum, and Urine.

Biofluids	Common biomarkers	Unique biomarkers	miRNA biomarkers	References
CSF	Aβ40, Aβ42, P-tau, T-tau, NFL	Neurogranin, BACE1, PSEN1, synaptotagmin, SNAP-25, GAP-43, synaptophysin, sTREM2, YKL-40, ICAM1, Secretogranin-2, Neuronal pentraxin 1, Neurofascin, Myelin basic protein, α-Synuclein, VILIP-1, Ferritin, hFABP, p-T181 tau, HSPA1A, NPEPPS, PTGFRN, FABP3, IL-6, IL-8, IL-10, MCP-1, MIP-1β	miR-342-3p, miR-125a-5p, miR-125b-5p, miR-451a, miR-23a-3p, ex-let-7i-5p, ex-miR-126-3p, ex-miR-151a-3p, miR-223, miR-29a, miR-193b, miR-342-3p, miR-3065-5p, miR-let-7d-5p, miR-let-7g-5p, miR-26b-5p, miR-191-5p, miR-125a-5p, miR-126-3p, miR-23a-3p, miR-151a-3p, miR-135b, miR-181a, miR-194-5p, miR-19b-3p, miR-29c-3p, miR-125b-3p, miR-31, miR-93miR-3613-3p, miR-3916, miR-4772-3p, miR-185-5p, miR-20b-3p, miR-501-3p, miR-545-3p, miR-181c, miR-139-5p, miR-141-3p, miR-150-5p, miR-152-3p, miR-23b-3p, miR-24-3p, miR-338-3p, miR-342-3p, miR-125b-5p, miR-342-5p, miR-1306-5p, miR-143, miR-15b, miR-15b-3p, miR-193b, miR-223, miR-451a, miR-29, miR-125b, miR-146a, miR-3065-5p, let-7i-5p, miR-106a-5p, miR-20-5p, miR-425-5p, miR-18b-5p, miR-582-5p, miR-106b-3p, miR-20b-5p, miR-146a-5p, miR-195-5p, miR-497-5p, miR-455-3p, miR-4668-5p, miR-5001-3p, miR-519, miR-548at-5p, miR-590-5p, miR-101-3p, miR-106b-5p, miR-143-3p, miR-335-5p,miR-361-5p, miR-138-5p, miR-155, miR-15a-5p, miR-659-5p, miR-93-5p, miR-101, miR-132, miR-212, miR-200c, miR-9, miR-30e-5p, miR-29a, miR-206, miR-142-5p, miR-384, miR-135a, miR-29b, MALAT1	Hansson et al., 2006; Lukiw, 2007; Ewers et al., 2008; Hu et al., 2010; Lukiw et al., 2012b; García-Ayllón et al., 2013; Dubois et al., 2014; Liu C. G. et al., 2014; Cheng et al., 2015; Dong et al., 2015; Kester et al., 2015; Lugli et al., 2015; Suarez-Calvet et al., 2016; Zetterberg et al., 2016; Rani et al., 2017; Bettcher et al., 2018; Molinuevo et al., 2018; Tariciotti et al., 2018; Wei et al., 2018; Dhiman et al., 2019; Jia et al., 2019; Ausó et al., 2020; Bǎlaşa et al., 2020; Knapskog et al., 2020; Muraoka et al., 2020; Zhuang et al., 2020; Pawlik and Blochowiak, 2021; Rastogi et al., 2021
Plasma	Aβ40, Aβ42, P-tau (p-T181 tau, P-S396-tau), T-tau	BACE1, PSEN1, TREM2, neurogranin, synaptotagmin, SNAP-25, GAP-43, synaptophysin, sTREM2, YKL-40, VILIP-1, HSPA1A, NPEPPS, PTGFRN, MIP-1β, cytokine I-309, interferon-γ, TNF-α, clusterin, TDP-43, YKL-40, BACE1, NFL, VILIP-1, sAPPβ, γ-secretase, soluble APP α, GDNF, GAP43, SNAP-25, synapsin 1, REST, IL-1, IL-4, IL-6, and IL-10, IL-8, IP-10, MCP-1, MIP-1β, cytokine I-309, Lamp 1, cathepsin D, ubiquitinylated proteins, HSP70, Synaptophysin, synaptopodin, synaptotagmin-2, neurogranin, Autolysosomal proteins, C1q and C4b, Factor D, Fragment Bb, C5b, C3b, C5b-C9, IRS-1, P-Serine-312-IRS-1, P-pan-tyrosine-IRS-1, HGF, FGF-2, FGF-13, IGF-1	miR-342-3p, miR-125a-5p, miR-125b-5p, miR-451a, miRNA23a-3p, ex-let-7i-5p, ex-miR-126-3p, ex-miR-151a-3p, miR-223, miR-29a, miR-193b, miR-342-3p, miR-3065-5p, miR-let-7d-5p, miR-let-7g-5p, miR-26b-5p, miR-191-5p, miR-125a-5p, miR-126-3p, miR-23a-3p, miR-151a-3p, miR-135b, miR-181a, miR-194-5p, miR-19b-3p, miR-29c-3p, miR-125b-3p, miR-31, miR-93, miR-3613-3p, miR-3916, miR-4772-3p, miR-185-5p, miR-20b-3p, miR-501-3p, miR-545-3p, miR-181c, miR-139-5p, miR-141-3p, miR-150-5p, miR-152-3p, miR-23b-3p, miR-24-3p, miR-338-3p, miR-342-3p, miR-125b-5p, miR-342-5p, miR-1306-5p, miR-143, miR-15b, miR-15b-3p, miR-193b, miR-223, miR-451a, miR-29, miR-125b, miR-146a, miR-3065-5p, let-7i-5p, miR-106a-5p, miR-20-5p, miR-425-5p, miR-18b-5p, miR-582-5p, miR-106b-3p, miR-20b-5p, miR-146a-5p, miR-195-5p, miR-497-5p, miR-455-3p, miR-4668-5p, miR-5001-3p, miR-519, miR-548at-5p, miR-590-5p, miR-101-3p, miR-106b-5p, miR-143-3p, miR-335-5p, miR-361-5p, miR-138-5p, miR-155, miR-15a-5p, miR-659-5p, miR-93-5p, miR-101, miR-132, miR-212, miR-200c, miR-9, miR-30e-5p, miR-29a, miR-206, miR-142-5p, miR-384, miR-135a, miR-29b, BACE1-AS, GAS5, has-Circ-0003391	Cogswell et al., 2008; Shioya et al., 2010; Lukiw et al., 2012a; Femminella et al., 2015; Goetzl et al., 2015b,^2016^; Jia and Liu, 2016; Sorensen et al., 2016; Winston et al., 2016; Zhang et al., 2016; Cosin-Tomas et al., 2017; Hara et al., 2017; Mullins et al., 2017; Wu et al., 2017; Kumar and Reddy, 2018; Yang et al., 2018; Cha et al., 2019; Fotuhi et al., 2019; Gamez-Valero et al., 2019; Goetzl et al., 2019; Kenny et al., 2019; Winston et al., 2019; Ausó et al., 2020; Bǎlaşa et al., 2020; Barbagallo et al., 2020; Liu L. et al., 2020; Pawlik and Blochowiak, 2021; Rastogi et al., 2021; Chen et al., 2022
Serum		BDNF, PHGDH	miR-31, miR-93, miR-143, miR-146a, miR-206, miR-135a, miR-193b, miR-384	Platenik et al., 2014; Dong et al., 2015; Goetzl et al., 2015a; Ausó et al., 2020; Yan et al., 2020; Rastogi et al., 2021
Saliva	Aβ40, Aβ42, T-tau, P-tau	Lactoferrin, AChE		Jann, 2000; Carro et al., 2017; Ausó et al., 2020; Bǎlaşa et al., 2020; Pawlik and Blochowiak, 2021
Urine	Aβ40, Aβ42, P-S396-tau	AD7c-NTP, MCP-1, ApoC3, SPP1, GSN, IGFBP7, Annexin2, Clusterin		Monte and Wands, 2001; Sancesario and Bernardini, 2019; Sun et al., 2019; Song et al., 2020; Watanabe et al., 2020; Xu et al., 2020; Rastogi et al., 2021

Aβ, Amyloid-beta; P-tau, phosphorylated-tau; T-tau, Total-tau; NFL, neurofilament light polypeptide; AChE, Acetylcholinesterase; BACE1, β-site amyloid precursor protein cleaving enzyme 1; PSEN1, Presenilin 1; GAP-43, Growth associated protein-43; TREM2, triggering receptor expressed on myeloid cells 2; sTREM2, soluble TREM2; YKL-40, Chitinase-3-like protein 1; ICAM1, intercellular adhesion molecule 1; VILIP-1, visinin-like protein-1; hFABP, heart-type fatty acid-binding protein; HSPA1A, Heat shock 70 kDa protein 1A; NPEPPS, Puromycin-sensitive aminopeptidase; PTGFRN, Prostaglandin F2 receptor negative regulator; FABP3, fatty acid-binding protein; MCP-1, monocyte chemoattractant protein 1; GSK-3β, glycogen synthase kinase 3β; DYRK1A, dual-specificity tyrosine-phosphorylation regulated kinase A; TNF-α, tumor necrosis factor α; TDP-43, transactive response DNA-binding protein 43; GDNF, glial-derived neurotrophic factor.

### Cerebrospinal fluid

CSF can be obtained by lumbar puncture, a diagnostic technique in which a needle is inserted into the subarachnoid space in the lumbar area (L3/L4 or L4/L5), securely below the spinal cord’s end.

Since CSF is in indirect contact with the brain’s extracellular space, biochemical changes in the brain are expressed in the CSF. In neurodegenerative diseases, neurovascular and blood-brain barrier dysfunction may occur. As a result, CSF is the best source of AD biomarkers (Molinuevo et al., 2014; Ferreri et al., 2021). T-tau, P-tau, and the 42-amino acid long amyloid beta peptide (Aβ42) are the most common AD CSF biomarkers.

In cognitively normal people, Aβ is formed in the brain and diffuses into the CSF, where it appears in moderate concentrations with multiple forms. Among them, the levels of Aβ42 and Aβ40 are relatively high. The former is a major component of amyloid plaques, which is regarded as an abnormal pathological lesion (Tiwari et al., 2019). The first step of amyloid pathogenesis is the generation of insoluble Aβ fibrils from amyloid precursor protein (APP) via cleavage. After oligomerization and subsequent polymerization, they transform into amyloid plaques (O’Brien and Wong, 2011). The toxicity of these plaques is associated with some mechanisms, including microglia infiltration, oxidative stress, and synaptic damage (Reiss et al., 2018).

The earliest studies of Aβ being secreted into the CSF date back to 1992, which encouraged researchers to develop corresponding immunoassays (Nitsch et al., 1992). Subsequently, decreased Aβ42 levels in the CSF of AD patients were revealed and validated, yet the cause was unknown for a long time (Gouras et al., 2015). In 2003, an autopsy study discovered a connection between low postmortem ventricular CSF Aβ42 levels and high plaque counts (Strozyk et al., 2003), a finding that was later confirmed in a study revealing a link between reduced CSF Aβ42 measured in ante mortem lumbar CSF samples and amyloid plaque counts measured autopsy (Tapiola et al., 2009). CSF Aβ42 levels can increase and then begin to decline 25 years before the onset of AD symptoms, according to a FAD cohort study from the Dominantly Inherited Alzheimer Network, while amyloid deposition measured with PET and Pittsburgh compound B, as well as an increased concentration of T-tau in the CSF, can be detected 15 years before expected symptom onset (Bateman et al., 2012). These results suggest that a decrease in CSF Aβ42 may be the first sign of preclinical Alzheimer’s disease. Low CSF Aβ42 levels have been linked to cognitive impairment in older women (Gustafson et al., 2007). CSF Aβ42 can also be used to predict cognitive impairment in healthy elderly people (Stomrud et al., 2007). In most cases, CSF Aβ42 and amyloid PET status have a high degree of concordance (Blennow et al., 2015). With sensitivity and precision of over 80%, a significant decrease in CSF Aβ42 and a significant increase in CSF t-tau and p-tau can be used to distinguish symptomatic AD patients (Blennow and Hampel, 2003). Hence, in 2012, the utility of CSF Aβ42 in predicting preclinical Alzheimer’s disease was verified in cognitively normal individuals with inherited AD genes.

In the brain (Suzuki et al., 1994), CSF (Portelius et al., 2006), and plasma (Zhang et al., 2018), Aβ40 is the most common source of Aβ peptide, although it does not appear to be as pathogenic as Aβ42 (McGowan et al., 2005). The CSF Aβ40 level in Alzheimer’s patients is higher than in patients with Parkinson’s disease dementia (PDD) or with dementia with Lewy bodies (DLB), implying that the Aβ42/Aβ40 ratio will help distinguish AD from PDD and DLB (Nutu et al., 2013). Also, the Aβ42/Aβ40 ratio could be used to differentiate AD from non-AD dementias better than CSF Aβ42 alone (Janelidze et al., 2016). The ratio of CSF Aβ42/Aβ40 is a better indicator of Aβ-positive PET than CSF Aβ42 alone (Pannee et al., 2016; Lewczuk et al., 2017), and is comparable to the ratios of t-tau/Aβ42 and p-tau/Aβ42 (Racine et al., 2016). Currently, fluid-based assays of Aβ40 and Aβ42 are commercially available.

Tau proteins are found in neuronal axons and play roles in preserving the cohesion of microtubules in neurons of the CNS. Tau proteins in the human brain are composed of six soluble isoforms and various phosphorylation sites (Khan and Bloom, 2016). Western blotting was used to classify tau proteins of Alzheimer’s disease in CSF in 1987 (Wolozin and Davies, 1987). The development of quantitative immunoassays made the measurement of tau proteins in CSF easier. ELISA method was used to quantify tau in CSF, this method was based on the sandwich format of a monoclonal anti-tau antibody against the mid-domain combined with a polyclonal anti-tau antiserum (Vandermeeren et al., 1993). Apart from acute brain trauma, T-tau CSF levels are emphatic, rising within days of injury and then remaining rapt for weeks before homogenization (Zetterberg et al., 2006). CSF T-tau levels are highest in diseases with the most severe neurodegeneration, such as Creutzfeldt–Jakob disease, Alzheimer’s disease, DLB, and frontotemporal dementia (FTD). Since it predicts more rapid clinical disease progression, CSF T-tau has been used as a biomarker for the severity of neurodegeneration (Buchhave et al., 2012; Hansson et al., 2018).

Tau is a phosphoprotein, indicating that its biological activity is completely regulated by its phosphorylation state (Gao et al., 2018). Highly phosphorylated tau proteins tend to form tangles rather than bind to microtubules, which may affect inter-synaptic signaling in brain neurons (Naseri et al., 2019). Since they were found in large numbers in neurofibrillary tangles in the brains of AD patients, they have been believed to be a reliable biomarker for AD (Lindwall and Cole, 1984; Bramblett et al., 1993). For different phosphorylated epitopes of tau proteins, ELISA methods have been established for threonine 181 and 231 (Blennow et al., 1995), threonine 181 (Vanmechelen et al., 2000), threonine 231 and serine 235 (Ishiguro et al., 1999), serine 199 [54], threonine 231 (Kohnken et al., 2000), and serine 396 and 404 (Hu et al., 2002). The phosphorylation state of tau in the brain is reflected in the concentration of phosphorylated tau protein in the CSF. The concentration of phosphorylated tau protein does not alter after an acute stroke, unlike the overall concentration of tau protein (Hesse et al., 2001). Sandwich ELISA was used to detect P-tau 181 in CSF in 2000, and it was discovered that levels were higher in AD patients relative to age-matched controls, but lower in FTD patients, indicating that CSF P-tau 181 may be a more specific marker for AD (Vanmechelen et al., 2000). Another sandwich ELISA for detecting P-tau threonine 231 was produced the same year, with 85 percent sensitivity and 97 percent specificity for distinguishing AD from non-AD controls (Kohnken et al., 2000).

Aβ42, T-tau, and P-tau levels are all considered practical biomarkers for AD (Lee et al., 2019). Moreover, the combined use of the three biomarkers in the diagnosis of AD has provided excellent results. For example, mild cognitive impairment (MCI) is the earliest clinical stage of AD, which is characterized by episodic memory problems. During clinical follow-up, MCI patients who progress to AD with dementia have the standard AD CSF biomarker profile (decreased CSF Aβ42 with increased T-tau and P-tau) already at this stage of the disease (Andreasen et al., 1999).

### Blood (plasma/serum)

Blood sampling would be preferable to CSF sampling when taking fluid samples to measure AD biomarkers, both for clinical diagnosis or screening and for repeated sampling in clinical trials, since blood is more accessible than CSF. However, blood-based biomarkers, on the other hand, encounter some challenges. Firstly, blood is a complicated fluid containing many cellular compartments as well as a constantly evolving environment of proteins, lipids, and other biochemical entities, which may intensify issues of reproducibility and necessitate concerted efforts to match methodology throughout studies (Thambisetty and Lovestone, 2010). Secondly, since the blood-brain barrier prevents molecules from freely passing between the CNS and the blood compartments, brain-derived biomarkers are found in low concentrations in the blood. Furthermore, certain biomarkers linked to Alzheimer’s disease pathology are expressed in non-cerebral tissues, which may make blood measurements difficult. Consequently, the quantities of brain proteins entering the blood must be calculated in a matrix containing extremely high levels of plasma proteins, posing a significant risk of analytical method intervention (Blennow and Zetterberg, 2015). Thirdly, brain proteins released into the bloodstream can be degraded by proteases, metabolized in the liver, or cleared by the kidneys, resulting in a variation that is unrelated to brain changes and difficult to control. Finally, heterophilic antibodies (endogenous antibodies which respond with the antibodies of the immunochemical test to measure the biomarker) can be present in the blood, resulting in falsely high or low performance. In CSF, where antibody levels are much lower, these types of antibodies are much less of a concern. Despite all the aforementioned issues that limit the possibility of discovering blood biomarkers for AD (O’Bryant et al., 2015), several biomarkers for AD diagnosis have been discovered.

The most widely studied blood markers for the diagnosis of symptomatic and prodromal AD are Aβ42 and Aβ40. Plasma Aβ42 levels are higher than plasma Aβ40 levels in AD patients at baseline and in those who develop AD within 3 years in a follow-up analysis (Mayeux et al., 2003). Individuals with elevated plasma Aβ42 had a more than twofold increased chance of AD onset relative to those with low plasma Aβ42 (Mayeux et al., 2003). A high plasma Aβ40 concentration has also been linked to an increased risk of dementia (van Oijen et al., 2006). Discovered that a lower plasma Aβ42/Aβ40 ratio is linked to greater cognitive impairment in elderly people without dementia over 9 years. The clinical stage of the examination and/or the combination of other forms of dementia can cause this dissimilitude. When compared to age-matched standard controls, Aβ42 levels are lower and Aβ43 levels are higher in the serum of AD patients, indicating that a lower Aβ42/Aβ43 ratio may be used as a blood marker for AD diagnosis (Zou et al., 2013).

Plasma tau concentrations, as determined by ultrasensitive assays, are higher in dementia patients than in cognitively normal controls, but not as significantly as in CSF (Pereira et al., 2021). Plasma tau has become a candidate blood marker for AD diagnosis due to the invasiveness and high costs of testing CSF tau, and several studies have concentrated on the quantitation of tau in AD, MCI, and normal classes. Since tau levels in plasma are much lower than in CSF (Karikari et al., 2020), ultrasensitive assays were created, which can be used to discover higher levels of T-tau in plasma from AD patients compared to control or MCI patients. However, there is no difference between MCI patients who developed AD and stable MCI patients.

Tau phosphorylated at threonine 181 (P-tau181) in blood has been widely recognized as a simple, accessible, and highly specific biomarker for AD diagnosis (Moscoso et al., 2021). Normally, plasma P-tau181 is higher in Alzheimer’s patients compared to people with normal cognitive and will increase as the disease progresses. Therefore, it may be used to estimate the likelihood of people AD in the future, regardless of disease stage (Brickman et al., 2021). Since the increase in plasma P-tau181 is related to Aβ and tau pathologies, it could also be utilized as a screening tool to study the pathophysiology of AD *in vivo* (Janelidze et al., 2020a). Of all the promising clinical applications for plasma P-tau181, the most important one might be to differentiate AD from other neurodegenerative disorders (Simrén et al., 2021). Another likely application for plasma P-tau181 is to determine whether patients need more detailed tau diagnostics testing with PET scans (Thebault et al., 2020).

Neurofilaments (Nfs) are the structural proteins of the neural cytoskeleton (Thebault et al., 2020). The constitutions of Nfs can be divided into three categories according to their molecular weight, which are Nf light (NfL), Nf medium (NfM), and Nf heavy (NfH) chains (Hendricks et al., 2019). Among the three subunits of Nfs, NfL is the most promising biomarker for the diagnosis of neurodegenerative diseases, which plays important role in neuron growth (Bacioglu et al., 2016). Because of the fairly low concentration of NfL in blood, it was not until the development of highly sensitive assays such as Simoa that researchers were able to perform NfL evaluations in blood (Mattsson et al., 2019). Dynamic changes in plasma NfL concentrations are correlated with neurodegeneration in AD, as Alzheimer’s patients had higher plasma NfL levels compared to healthy individuals (Mattsson et al., 2017; Preische et al., 2019). While plasma NfL cannot be used to distinguish AD from other dementias, it may serve as a practical tool for neurodegeneration detection. In addition, it could be used in combination with other available AD biomarkers for AD diagnosis (Blennow and Zetterberg, 2018b).

### Urine and saliva

Urine is a desired substitute for blood since it can be collected in large quantities easily and non-invasively (Hartmann and Kist, 2018). Although the composition of organic compounds in urine is less complex than that of CSF and blood, changes in metabolites and hormones among these compounds can reflect the metabolic and pathophysiological state of a person (Sancesario and Bernardini, 2019). At the same time, the relatively low compositional complexity means that fewer proteins capable of interfering with measurements are present in the urine (Bǎlaşa et al., 2020; Buccellato et al., 2021). Some AD biomarkers have already been identified in urine.

Alzheimer-associated neuronal thread protein (AD7c-NTP) has attracted a great deal of attention from researchers because of the correlation between its concentration changes in urine and the severity of AD (Wang et al., 2019; Ausó et al., 2020). Elevated urinary AD7c-NTP levels can be used in the early diagnosis of AD with high accuracy (Li Y. et al., 2020; Seol et al., 2020). Monocyte chemoattractant proteinx (MCP-1) also showed promise as a biomarker for AD. Its concentration in the urine of AD patients was discovered to be significantly higher than that of cognitively normal individuals (Xu et al., 2020). In addition, Watanabe et al. (2020) found that the AD group had significantly higher levels of apolipoprotein C3 (ApoC3) in urine than the control group did. Therefore, it could be utilized to differentiate patients with AD. Recently, Yao et al. (2018) applied a combination of computational and experimental methods to identify three differentially expressed proteins. To be specific, in the urine of AD patients, secreted phosphoprotein 1 (SPP1) was downregulated, whereas gelsolin (GSN) and insulin-like growth factor-binding protein-7 (IGFBP7) were upregulated, which made them promising biomarkers for AD.

In addition to the aforementioned proteins, several indicators of other disorders in urine can be considered candidates to be biomarkers for AD early detection. For example, the levels of formaldehyde and 8-hydroxy-2′-deoxyguanosine (8-OHdG) can be used to access the status of formaldehyde metabolism and oxidative DNA damage, respectively. They were significantly higher in the urine of AD patients compared to cognitively normal controls (Pena-Bautista et al., 2019; Wang et al., 2022). AD would interact with osteoporosis during its progression. Pu et al. (2020) found that urinary biomarkers reflecting osteoporosis and bone loss, such as deoxypyridinoline (DPD), creatinine (Cr), and calcium (Ca), can also be used in the early diagnosis of AD in male populations.

Similar to urine sampling, saliva sampling is non-invasive, stress-free, and easy to obtain (Lee et al., 2019; Veerabhadrappa et al., 2020; Francois et al., 2021). The origin of AD-related molecules in the saliva is unknown. It is speculated that they are secreted by the salivary glands or transported from the blood (Schepici et al., 2020). In addition, the flow and composition of saliva are directly regulated by the glossopharyngeal nerve and the facial nerve (Farah et al., 2018; Pawlik and Blochowiak, 2021). Given their proximity and relation, biomarkers may be secreted from the nerves into the salivary glands (Gleerup et al., 2019; Paraskevaidi et al., 2020). These characteristics may render saliva a promising matrix for biomarker discovery. Although saliva has quite a few unique advantages, the relatively low concentration of analytes in saliva makes detection more difficult (Reale et al., 2020). In recent years, developments in detection methods have provided a tempting opportunity to use saliva to diagnose diseases (Ashton et al., 2019). A growing number of researchers have been working to explore the association between salivary biomarkers and AD.

In exploring urinary biomarkers for AD diagnosis, many researchers have focused on Aβ42 quantification. A collection of studies has illustrated the potential use of salivary Aβ42 as a diagnostic tool for AD. Bermejo-Pareja et al. (2010); Kim et al. (2014), and Sabbagh et al. (2018) found that the salivary Aβ42 was significantly higher in AD patients than in healthy participants, although controversy exists regarding changes in Aβ42 secretion with the development of AD. In addition, according to the study by McGeer et al. (2018) salivary Aβ42 levels can be used to predict the likelihood of future AD onset. Furthermore, Lee et al. (2017) revealed that salivary Aβ42 concentrations in patients with Parkinson’s disease were identical to those in healthy subjects, indicating that Aβ42 was specific for AD. However, Shi et al. (2011) and Lau et al. (2015) found that Aβ42 in saliva was undetectable, whereas the finding of Tvarijonaviciute et al. (2020) showed that salivary Aβ42 levels were significantly lower in AD patients than in controls. The inconsistency between these experimental results may be due to differences in saliva collection, storage, and testing methods, as well as differences in the subjects screened.

Lactoferrin is an iron-binding glycoprotein that is abundantly expressed in saliva. It has been considered a potential biomarker for AD because of two properties, namely anti-microbial and Aβ-binding (Zhang et al., 2021). Carro et al. (2017) measured salivary lactoferrin levels in some healthy individuals, patients with AD, patients with MCI, and patients with PD. The results showed that salivary lactoferrin concentrations were highest in PD patients, while patients with AD or MCI had lower salivary lactoferrin concentrations than the controls did. Low lactoferrin concentration in saliva was hypothesized to be associated with the risk of developing AD or MCI. In addition, Gonzalez-Sanchez et al. (2020) found that participants with FTD did not experience a significant decrease in salivary lactoferrin concentrations. These two studies demonstrate the value of this biomarker in diagnosing the early clinical stages of AD.

Glial fibrillar acidic protein (GFAP) is a component of the cytoskeleton of astrocytes, which plays an important role in modulating neuronal inflammation (Yang and Wang, 2015). According to the study of Katsipis et al. (2021) two different immunological assays, namely ELISA and Dot Blot, were applied to detect GFAP levels in the saliva of participants in the experiment. The results obtained by both methods showed a decrease in GFAP levels in patients with AD or MCI compared to healthy subjects. In addition, GFAP levels in the saliva of AD patients are significantly lower than those of MCI patients. The possible reason for this was that GFAP were susceptible to several post-transcriptional modifications, which would hinder their transfer from the brain to the saliva and reduce their solubility (Katsipis et al., 2021). Since salivary GFAP can be used to differentiate between healthy individuals, people suffering from MCI, and AD patients, it may present as a reliable biomarker for the screen of the neurological status of people.

Although researchers have uncovered these urinary and salivary biomarkers that are of value for the early diagnosis of AD, there is still much work to be done in the future. Firstly, standards for sampling, processing, storage, and analysis methods need to be developed (Bouftas, 2021; Goldoni et al., 2022). Secondly, before these novel biomarkers can be in the clinic, large-scale as well as longitudinal research is needed to further determine their reliability in detecting AD pathology (Liang and Lu, 2019). Finally, to test their specificity for AD, it is imperative to study the levels of the biomarkers in other neurodegenerative conditions (Ashton et al., 2021).

### Exosomes (extracellular vesicles)

**FIGURE 5:**
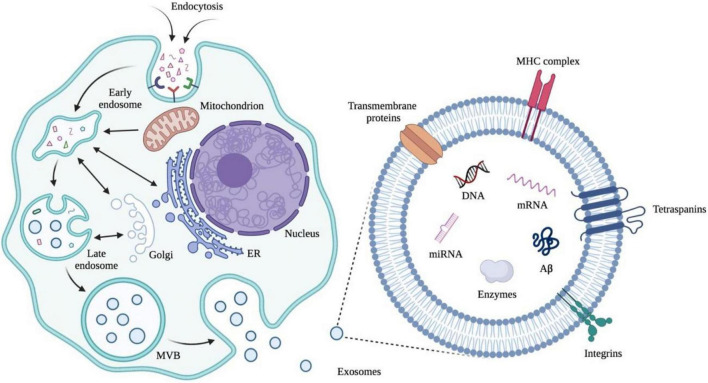
Early endosomes are generated from extracellular cargos as well as cell surface proteins through endocytosis. Mitochondria, endoplasmic reticulum (ER), and Golgi are involved in the formation of multivesicular bodies (MVBs) from early endosomes through fusion. MVBs can fuse with plasma membranes and release exosomes, which are membrane nanovesicles that contain plasma-membrane- and cytosol-associated molecules such as nuclear acids and proteins both inside and outside the vesicles. Illustrations were generated using BioRender.

The application of exosomes derived from human cells to examine an individual’s health status is an emerging molecular diagnostic (Figure 5, Trams et al., 1981; Johnstone et al., 1987; Sokolova et al., 2011; van der Pol et al., 2014; Greening et al., 2015; Pegtel and Gould, 2019). The focus of the published papers is related to cancer to parse the roles of exosomes in pathophysiology, autophagy, diagnosis, and therapeutic applications (Colletti et al., 2020; Jafari et al., 2021; Kugeratski and Kalluri, 2021; Lakshmi et al., 2021; Liu T. et al., 2021; Sandua et al., 2021; Xu et al., 2021; Zhou et al., 2021). As the exosomes are also synthesized and released from brain cells to blood or CSF through the BBB, alterations in the contents of exosomes may serve as biomarkers to detect the pathological progress of the CNS (Kanninen et al., 2016; Jiang et al., 2019; Vandendriessche et al., 2020; Pinnell et al., 2021). Structurally, exosomes are rich in certain tetraspanin proteins such as CD81, CD82, CD37, and CD63, which mediate the other protein inclusion, including immunoglobulin superfamily member 8 (IGSF8) major histocompatibility complex (MHC) class II proteins, syndecans (SDC1–4) and integrins (Raposo et al., 1996; Escola et al., 1998; Hemler, 2003; Segura et al., 2005; Liang et al., 2014; Kalluri and LeBleu, 2020). Notably, a growing number of studies certify that some inclusions in exosomes are RNAs in functional form with the ability to influence other cells and tissues (Ratajczak et al., 2006; Valadi et al., 2007; Skog et al., 2008; Pegtel et al., 2010). In addition, the exosomes cover a variety of DNAs which can be used to infer tissue-of-origin and molecular classification (Balaj et al., 2011; Kahlert et al., 2014; Thakur et al., 2014; Sansone et al., 2017). Taken together, exosomes have the potential to expand the central paradigm of liquid biopsy for preclinical AD diagnosis and the biomarker library (Yin et al., 2020; Guo et al., 2021).

In addition to biomarkers, clinical, physiological, and neuropsychological tests are also an important part of detecting clinically symptomatic persons. However, waiting periods for specialist appointments in hospitals can be very lengthy, causing severe and sometimes life-threatening delays for AD patients (Patterson et al., 2004). Besides, due to a lack of experience and skill, cognitive tests are often performed, scored, and interpreted inappropriately in primary care centers (Cannon and Larner, 2016), which gives rise to differences in diagnosis, evaluation, and assistance between primary care and specialty care systems (Garcia-Ptacek et al., 2017). Consequently, both physicians and patients will benefit a lot from a relatively simple first-line test determining which patients require more detailed diagnostics testing by measuring biomarkers in body fluid. Such a procedure would reduce the number of needless referrals and diagnostic tests and thus decrease the total pressure on healthcare systems (Hampel et al., 2017; O’Bryant et al., 2017). Furthermore, improvements in AD diagnosis produced by liquid biopsy could in turn promote symptomatic treatment (Hampel et al., 2021).

## Conclusion

Undoubtedly, fluid-based biomarkers are crucial in the preclinical diagnosis of AD. For patients with cognitive problems, particularly those with mild memory loss as the only objective symptom, the current diagnostic method is incapable of predicting whether the end is Alzheimer’s disease or not (Tan et al., 2014). Having outlined a huge collection of the literature, a good deal of analytes that had been claimed to have the diagnosis value for AD were revealed. As we summarized above, accompanying the theory innovation, the liquid biopsy technologies including the equipment and kits emerge in endlessly. However, the practice has proven that the relentless efforts worldwide have not reached a satisfactory conclusion. In other words, no one strategy has “saved” the doctors and researchers from the current plight. The cause is varied and super-complicated.

First of all, the etiology of AD has many risk factors or heterogeneity, including but not limited to smoking, drinking, aging, education, and underlying medical conditions (obesity, smoking, diabetes, hypertension, and cardiac disease). Importantly, the principal cause has not been deduced. Even the well-established suspicious loci only account for 15∼20% of the AD pathology, which was often obtained from population-based studies and taken as the fluid biomarkers. As a result, the model in mathematical statistics is hard to build because of too many variables, especially the unknowns.

Secondly, none of the current technologies in liquid biopsy can perfectly meet the clinical demand. On one hand, the CSF biomarkers Aβ42, total tau, P-tau181, P-tau217, and P-tau231 show a high diagnostic accuracy for AD, even for prodromal AD patients with MCI. Therefore, they are increasingly being used in the clinic and valued as a similar gold standard. However, the procedure for acquiring CSF is expensive and the individuals should suffer a lumbar puncture and extra risk. On the other hand, fluid-based biomarkers significantly boost AD diagnosis by lowering expense and increasing ease of testing. As opposed to CSF markers, they may provide a minimally invasive, time- and cost-effective method of early detection and diagnosis of AD (Olsson et al., 2016). Taking the three aforementioned phosphor-tau in blood as an example, only in specific scenarios, they can show good performance by independent use.

Thirdly, several joint diagnostic strategies have been applied to reveal the full view of AD pathologies. The biomarker-based amyloid-tangle-neurodegeneration (A-T-N) framework proposed by Jack et al. (2016) and updated by NIA-AA (Jack et al., 2018) can capture the whole spectrum pathophysiological features of AD. By which, AD can be distinguished from other neurodegenerative diseases. Even so, in this scheme, not all of the pathologies and related biomarkers are included. Therefore, Huang et al. (2022) suggested adding an X to form the A-T-N-X framework, focusing on the CNS and periphery biomarkers associated with inflammation, systemic immunity, metabolism, and synaptic damage, neuroinflammation, glial cells. In the mid-to-long-term future, these frameworks should be validated in many clinical trials.

Lastly, many studies predict that faulty autophagy occurs before the formation of the fluid biomarkers such as Aβ or senile plaques, in the development of AD (Li W. et al., 2020; Lee et al., 2022). If in that case, the overwhelming majority of the aforementioned biomarkers cannot be employed in the routine clinical diagnostic for the very early stage of AD.

For now, it is foreseeable that an increasing number of blood-based biomarkers would be incorporated into diagnostic criteria and research frameworks for AD, in combination with current CSF biomarkers, to improve the preclinical and clinical diagnosis of AD, the world’s most common age-related neurodegenerative disease.

## Author contributions

MT and HTZ designed the study. JL performed the data analysis. YL and XG wrote the manuscript All authors made a direct and intellectual contribution to this topic and approved the article for publication.

## Footnotes

1
https://www.sysmex.co.jp/en/rd/report/index.html
